# Current Status of Fertility Preservation in Pediatric Oncology Patients

**DOI:** 10.3390/children11050537

**Published:** 2024-04-30

**Authors:** Albert Pasten González, Cristina Salvador Alarcón, Jaume Mora, Marta P. Martín Gimenez, Rosalia Carrasco Torrents, Lucas Krauel

**Affiliations:** 1 Pediatric Surgical Oncology Unit, Department of Pediatric Surgery, SJD Barcelona Children’s Hospital, Universitat de Barcelona, 08950 Barcelona, Spain; apastego44@alumnes.ub.edu (A.P.G.); martapilar.martin@sjd.es (M.P.M.G.); rosalia.carrasco@sjd.es (R.C.T.); 2 Pediatric Cancer Center Barcelona, SJD Barcelona Children’s Hospital, Universitat de Barcelona, 08950 Barcelona, Spain; jaume.mora@sjd.es; 3 Department of Obstetrics and Gynecology, SJD Barcelona Children’s Hospital, Universitat de Barcelona, 08950 Barcelona, Spain; cristina.salvador@sjd.es

**Keywords:** fertility preservation, cancer, pediatrics

## Abstract

Cancer poses significant emotional challenges for children and adolescents, despite improvements in survival rates due to new therapies. However, there is growing concern about the long-term effects, including fertility issues. This review examines recent advancements and future directions in fertility preservation within a pediatric population subjected to oncological therapies. Worldwide, there is variability in the availability of fertility preservation methods, influenced by factors like development status and governmental support. The decision to pursue preservation depends on the risk of gonadotoxicity, alongside factors such as diagnosis, treatment, clinical status, and prognosis. Currently, options for preserving fertility in prepubertal boys are limited compared to girls, who increasingly have access to ovarian tissue preservation. Adolescents and adults have more options available, but ethical considerations remain complex and diverse.

## 1. Introduction

Cancer is a devastating illness, impacting not only the patients but also their family, especially when it afflicts a child or adolescent. Each year, approximately 200,000 patients under the age of 15 are diagnosed, constituting 2% of all new cancer cases [1,2]. In the last five decades, there has been a significant rise in survival rates, escalating from 20–30% in the 1960s to 80% presently. This remarkable improvement can be attributed largely to worldwide collaborative endeavors [3,4] and the adoption of new therapies like chemotherapy, radiotherapy and/or surgery. However, it is important to note that these treatments, while beneficial, can also have side effects, including potential impacts on future fertility.

For many adults, parenthood is a significant life goal. However, individuals who have battled childhood cancer may face obstacles in achieving this goal due to the toxic effects of their treatment [5]. This issue has garnered increasing attention in recent times, leading to a surge in research endeavors in pediatric patients that have contributed to deepening our comprehension of protective methods and the transfer of cryopreserved tissue. Studies focusing on preservation techniques affirm the safety of surgically retrieving gonadal tissue (both ovarian and testicular) for cryopreservation. However, while there is a clear pathway for utilizing ovarian tissue, the same is not true for testicular tissue, posing a current challenge in this area of research [6]. Traditionally, fertility preservation options have been more readily available to adults, whereas children have had limited access to such options [6,7], attributable to factors like insufficient professional expertise, time constraints, and the urgency to commence treatment promptly [8]. The overarching objective should be to provide fertility preservation opportunities to every child undergoing cancer treatment [7,9,10,11].

This review aims to succinctly summarize and analyze the latest scientific evidence concerning fertility preservation in pediatric cancer patients, shedding light on the current landscape and future challenges within this realm of pediatric cancer care.

## 2. Methods and Results

### 2.1. Present Situation

Since the first successful birth from in vitro fertilization in the late 1970s, significant progress has been achieved in the field of fertility preservation. Procedures such as freezing oocytes, embryos, and sperm have demonstrated favorable outcomes in fertile adults [12]. These techniques have traditionally been employed for reasons such as postponed parenthood, research purposes [13], gender reassignment, or addressing autoimmune or genetic conditions affecting fertility [11].

Many countries have taken steps to ensure universal access to fertility preservation for children. Suzuki et al. [14] discuss the development of Japanese guidelines for child fertility preservation, drawing from both domestic experiences and international initiatives. Robson et al. have examined the current state of affairs in Australia, revealing disparities in access across different health-care facilities, with up to 26% of families unable to access preservation services due to financial constraints [15].

Renowned European institutions, such as those in Edinburgh [16], Israel [17], and Belgium [18], have established comprehensive fertility preservation programs, with notable achievements including the first successful births from transplanted cryopreserved ovarian tissue in Belgium. Switzerland has also established a successful multicenter network for cryopreserving testicular tissue [19]. Sweden has offered fertility preservation techniques since the late 1980s, with coverage extended universally through the country’s health-care system [13]. Similarly, Portugal has adopted a national policy ensuring access to fertility preservation, accompanied by professional training initiatives and informational resources for patients and families [20]. Spain has recently released a national multicenter and multidisciplinary consensus on fertility preservation [21]. All of these national programs and public policies are designed for the general population, sometimes including children [13,16,19].

In 2018, Oktay et al. [9] updated the clinical practice guidelines of the American Society of Clinical Oncology (ASCO), affirming ovarian tissue cryopreservation as a clinically viable option for both adult women and girls without the necessity for prior hormonal stimulation. However, they underscored that testicular tissue preservation remains experimental, necessitating further research before clinical implementation.

Mulder et al. have published recommendations based on systematic reviews for children, adolescents, and young adults (CAYAs) diagnosed with cancer aged 25 years or younger. These recommendations, prepared by a multidisciplinary group on behalf of the European research project PanCareLIFE in collaboration with the International Late Effects of Childhood Cancer Guideline Harmonization Group (IGHG), aim to improve knowledge and practices concerning the long-term follow-up of childhood cancer survivors [10,22].

### 2.2. Risks to Future Fertility

Patients diagnosed with cancer often face a spectrum of therapies that may jeopardize immature or mature germ cells [7]. Historically, when treatment options for achieving a cure were limited, the primary emphasis was on survival, leaving little room to consider potential consequences such as infertility [1]. As survival rates improved, it became evident that cured adults experienced sequelae such as second malignancies, pulmonary fibrosis, heart disease, and varying degrees of infertility. Consequently, a risk-adjusted approach emerged, tailoring therapy to each cancer type, maintaining comparable cure rates while minimizing adverse effects, particularly in patients requiring more intensive treatments [4].

These comparisons have enabled the identification of treatments most likely to impact future fertility, a concept termed gonadotoxicity. Up to 20–30% of childhood and adolescent cancer survivors have been affected to some extent by gonadotoxicity [7].

### 2.3. Chemotherapy

The gonadal damage caused by chemotherapy, particularly alkylating agents [23], which form covalent bonds with DNA, breaking the double strand and causing cell death, is well known. This produces an antineoplastic effect, but also immediate toxic effects, such as bone marrow aplasia and long-term sterility. Examples of this group include cyclophosphamide, procarbazine, and platinum derivative complexes such as cisplatin. The latter are less likely to affect fertility [24].

Gonadotoxicity occurs in both female and male patients, although the risk is higher in the latter because girls are protected by their high reserve of primordial follicles [25].

One way to estimate risk is to use the cyclophosphamide equivalent dose (CED) (Table 1). This score is calculated by adding the doses of the drugs given to the patient over the course of treatment, multiplied by certain factors [26].

### 2.4. Radiation Therapy

Radiation therapy is a fundamental part of cancer treatment and has been used for more than a century. It works by ionizing molecules, removing their electrons to cause changes in them. When DNA is ionized, its replication is inhibited and cells die, especially if they are multiplying rapidly. Current technology allows the treatment to be delivered locally, eliminating the need to irradiate large areas of the body.

In girls, pelvic radiation causes permanent gonadal damage, which increases with age at the time of radiation as ovarian reserve decreases (Table 2) [27]. In addition, the uterine tissue and its vascularization are affected by radiation and become resistant to hormone replacement therapy. This effect has been described particularly in prepubertal girls [28].

In boys, a transient decrease in sperm count has been described with radiation doses of 2–4 Gy, and no spermiogram recovery has been reported with doses greater than 12 Gy. Doses greater than 24 Gy are associated with cessation of puberty, requiring the initiation of hormone replacement therapy [27].

### 2.5. Other Interventions

The third pillar of cancer treatment is surgery. Sometimes, the gonads must be removed due to primary or metastatic neoplastic involvement, either bilaterally at diagnosis or at metachronous time. Examples include germ cell tumors or leukemic or neuroblastoma infiltration.

Hematopoietic stem cell transplantation (HSCT) is a technique used for a diverse group of pathologies, not only neoplastic, and its performance in most pediatric transplants requires prior use of chemotherapy or myeloablative chemoradiotherapy, with the risk of compromising future fertility [24].

### 2.6. Indications for Fertility Preservation

Once the inherent risk associated with the oncological pathology and its treatment has been assessed (see Table 3), additional factors must be taken into account to determine the necessity for fertility preservation. In cases where the risk of gonadotoxicity is high, there should be no hesitation, and the procedure should be carried out promptly. However, when the risk is deemed intermediate or low, factors such as the patient’s prognosis, age, and clinical condition should be included in the discussion regarding the indication for these procedures [11,12,29].

In certain instances of cancer, when the initial treatment did not involve gonadotoxic therapy, but the disease proves refractory or relapses necessitating more intensive therapy, secondary or salvage fertility preservation may be considered.

### 2.7. Used Techniques

#### 2.7.1. Prepubertal Children

This group is arguably the most intricate due to the experimental nature of the procedures conducted thus far, rendering it a highly dynamic area of research [19,30,31].

Several ways have been proposed to restore fertility from immature testicular tissue. One of them is the intratesticular injection of autologous spermatogonial stem cells to repopulate the seminiferous tubules [32]. This is a complex procedure that requires great knowledge of human testicular anatomy, since the best injection site is the rete testis, which is not in the same location as in experimental animals, and inoculation of the cells into the gonadal interstitium will cause the technique to fail. Issues still to be resolved include the number of injections required, the volume and speed of infusion so as not to damage the tubules [33], and how to help the cells migrate to the seminiferous tubules, where they must adhere to the basal membrane to begin their replication and maturation [32].

Another possibility is autologous transplantation of testicular tissue [34], which maintains the paracrine microenvironment for spermatogenesis provided it is implanted in the scrotum. To date, only in vitro spermatogonial maturation has been achieved in animals and normal offspring in pigs and rhesus monkeys. Spermatogenesis of prepubertal human testicular tissue has also been achieved when implanted in monkeys. While the results are promising, there are still some issues to be resolved: the appropriate volume of tissue to implant, the optimal age for transplantation, and the appropriate timing of sperm retrieval [30]. There is also a latent fear of reimplantation of viable tumor cells that could infiltrate the testicular tissue, especially in pathologies that can affect the testis, such as leukemia, lymphoma, or neuroblastoma [35].

The third method described is in vitro maturation of spermatogonia for subsequent intracytoplasmic injection into oocytes by culturing testicular tissue or cell suspensions introduced into a 3D matrix simulating seminiferous tubules [34]. To date, sperm development has been achieved in animals, and healthy offspring have been obtained in monkeys [30]. Tesarik et al. in 1999 reported the birth of three children from spermatozoa obtained in vitro, but this work was highly criticized because the results could not be replicated and it was suggested that these spermatozoa could have been derived from haploid cells already present in the cultured sample, since the tissue they worked with was adult [30]. Tissue culture has been the most successful method because it preserves the microarchitecture in which spermatogonia normally proliferate and develop. The problems to be solved are the ideal culture medium, appropriate temperatures, and other conditions [34]. Regarding suspension culture, the aim is to find the appropriate matrix that promotes the cellular organization of spermatogonia, both from animal and synthetic models [36]. Gametogenesis has been achieved in matrices, but with adult testicular tissue [30,34]. It is known that the extracellular matrix and supporting cells (Leydig, Sertoli) are very important in promoting gametogenesis, so studies are also considering how to promote their proliferation in developing matrices [37].

#### 2.7.2. Adolescents and Young Adult Men

In this group, fertility is preserved by freezing a semen sample [7]. There are several difficulties, such as from what biological moment does semen has enough spermatozoa and what to do if the patient is unable to masturbate.

In the first case, it was difficult to establish specific age or developmental criteria. It has been reported that samples from children as young as 11 years of age are suitable for use [25], and semen cryopreservation is generally indicated in adolescents with Tanner 3 pubertal stage and older, despite variability in sexual maturation compared with secondary sexual characteristics.

In the second case, alternatives to masturbation can be offered, such as ejaculation using stimulators that can be used while the patient is under anesthesia for any other procedure. Another option is testicular sperm extraction by microsurgery, which can also be performed under general anesthesia [13,25].

#### 2.7.3. Prepubertal Girls

In recent years, ovarian tissue cryopreservation has become a clinical option and the only alternative for prepubertal girls [38]. More than 130 successful births have been reported with this technique, and restoration of hormonal function has been achieved in up to 95% of cases [39]. Ovarian stimulation is not necessary to obtain the samples, and the amount of tissue to be obtained varies according to age and ovarian size: in young girls or those at high risk of infertility, oophorectomy is recommended [16], although there is no standard amount of tissue that can be established [29]. Sampling may be performed under general anesthesia for other reasons, and it is not contraindicated to collect ovarian tissue if chemotherapy has already been started [40,41].

To rule out minimal residual disease, especially in patients with leukemia, neuroblastoma, and Burkitt’s lymphoma, some of the excised tissue is sent for pathology analysis. Other screening methods include immunohistochemistry, immunofluorescence, or polymerase chain reaction (PCR) [18,39]. It has been reported that up to 30% of ovarian samples taken from leukemia patients could have some degree of minimal residual disease using these screening tools, but the potential to produce a relapse after reimplantation is unknown [42]. To further minimize the risk, in vitro–ex vivo follicle culture techniques (i.e., in the gonadal sample already obtained) have been described for freezing together with the tissue and in vivo implantation of previously obtained follicles in an artificial ovarian matrix [18].

If necessary, the tissue is reimplanted, which can be done orthotopically or heterotopically. In the former, the tissue is placed on the remaining atrophic ovary or in the contralateral ovary. In the latter, it is placed in a retroperitoneal pocket or in the subcutaneous tissue of the forearm [25]. Most post-reimplantation pregnancies have been described in the orthotopic form [43], although there are cases of pregnancies obtained from oocytes generated in heterotopic sites [24]. Spontaneous resumption of menstrual cycles has been reported 6–8 months after reimplantation; however, hormonal activity of FSH and estradiol is already present after 4 months [24].

In cases of pelvic radiotherapy, there is the possibility of gonadal transposition, in which the ovaries are freed by laparoscopy and fixed as far as possible from the field to be irradiated [44]. This technique is also useful for the testicles, which may be located in the inguinal region.

#### 2.7.4. Adolescent and Young Adult Women

Oocyte cryopreservation is the most widely used technique and requires a cycle of ovarian stimulation with ultrasound monitoring of follicles for subsequent transvaginal retrieval [25].

This procedure is widely known and accessible; however, it has some disadvantages. Patients without previous sexual contact are heavily invaded by transvaginal procedures. Moreover, ovarian stimulation involves the need to delay oncological treatment [7,9,39]. However, it is possible to plan ovarian stimulation in postpubertal women who have not had sexual contact, since follicular monitoring is done by abdominal ultrasound and oocyte retrieval can be performed during anesthesia for another procedure. Indication must be assessed on a case-by-case basis.

The idea of ovarian suppression as a method of fertility preservation by administration of GnRH has been explored, but the clinical results are contradictory [18]. However, it is still recommended when other preservation techniques are not available [45].

#### 2.7.5. Preventive Measures and Advocating for Gonadal Preservation in Benign Tumors

Beyond the traditional approach of gonadal transposition, gonadal shielding during imaging and radiotherapy for minimizing radiation exposure to the gonads while maintaining diagnostic accuracy and therapeutic efficacy is widely adopted.

The paradigm of gonadal preservation extends beyond malignant conditions to encompass benign tumors of the testes and ovaries. Benign lesions may necessitate surgical intervention, thereby posing a risk to gonadal integrity. By advocating for gonadal-sparing procedures in these cases, clinicians can mitigate the risk of iatrogenic gonadal dysfunction without compromising therapeutic outcomes. Moreover, such an approach aligns with the principles of patient-centered care, emphasizing fertility preservation and quality of life as key priorities.

### 2.8. Ethical Considerations

The survival rate of children and adolescents with cancer is increasing and obliges us to provide the care that these patients need once they have overcome the disease. In this sense, fertility preservation is an aspect that should be considered very carefully by the centers that take care of this type of patient [46], because many of the surviving children will grow up and probably want to have natural children [5].

The first challenge is to ensure that the professionals involved have the necessary information and knowledge on the subject. This will allow them to set up circuits to inform, support, and help the patient and his/her family to make a decision, without delaying the start of oncological treatment [20,47]. As far as possible, referral alternatives should be sought if the treatment center does not have a human reproductive unit [8]. This lack of systematization and lack of established internal and external referral pathways leads to less discussion, fewer referrals, and as a result, many patients with indications for fertility preservation lose their possibility of saving gametes and/or tissue [48].

The second challenge is to consider and manage potential ethical conflicts, such as the autonomy of a patient who cannot exercise it. Parents or caregivers may refuse fertility preservation because of fear, ignorance, religious beliefs, or because they are overwhelmed by an oncological diagnosis, thus depriving the child or adolescent of the one chance of preserving their fertility. It is important to be close and warm, to provide a calm environment for discussion, to have circuits that allow rapid action once consent is obtained, and to have educational materials to facilitate the process [49].

Other ethical conflicts to consider may be the risk of reimplantation of disease in certain cases, as noted above with ovarian cryopreservation, or the possibility that the patient may die or be incapacitated, and his or her gonadal tissue samples or gametes remain available [50]. These issues should be informed and discussed with great care, avoiding fear or rejection, and legal assistance should be sought in drafting informed consents or informing parents, if necessary [8]. Cost is also an important issue to address, as it is unacceptable for a patient to lose the opportunity to preserve fertility due to lack of money [15].

## 3. Discussion

Fertility preservation and pediatric oncology are two worlds that have gradually met. Over the years, concern for the integral well-being of long-term survivors has grown and technology has advanced to offer new therapeutic alternatives.

In children, the priority has always been disease-free survival, initially at any cost. Now, the adaptation of therapy according to risk [1,3,4] has made it possible on the one hand to better stratify the treatment to be administered and on the other hand to give more importance to the quality of life of the surviving patient. One of the factors that affects the quality of life of almost everyone is having options for future parenthood, if desired, and we must offer options to every childhood cancer survivor [7].

Coordination between the different disciplines involved in the diagnosis, treatment, and follow-up of children is of paramount importance, as there is little time to initiate therapy, especially in hematological pathologies, so it is essential to do things quickly and well.

Although it has been known from the beginning that cancer treatment is potentially toxic and produces side effects, it is only in the last few decades that this has become important [26]. This has made it possible to define which patients are at risk of compromising their fertility. Alkylating agents such as cyclophosphamide and platinum derivatives are most strongly associated with this risk. On the other hand, radiotherapy also has a direct detrimental effect on the gonads [27], and various surgical procedures can compromise the partial or total viability of the gonads.

Taking this into account, the indications for fertility preservation are evaluated according to the risk of gonadotoxicity, the clinical condition of the patient, and his vital prognosis [44].

Prepubertal males are the only group for which there is no clinically effective method of fertility preservation [31,35]. Experimental studies are underway to preserve testicular tissue for future reimplantation [34,35], to mature previously harvested spermatogonia for intracytoplasmic injection into oocytes [30], and to inject spermatogonial stem cells intratesticularly to recolonize the seminiferous tubules [32].

In adolescents and adults, sperm freezing is a simple method of fertility preservation [7]. The complexity lies in the fact that sometimes masturbation is not possible due to age or religious/social considerations. In these cases, there are ways to obtain semen samples with the child under anesthesia, electrostimulation, or microsurgery while another procedure is being performed [25].

Ethical aspects considered in this matter relate to the ability to offer these procedures to any patient who requires them in a flexible and secure way, the management of the principle of autonomy of patients who have no legal capacity, and ethical dilemmas such as the final disposal of samples in the event of death. It is important that these issues are considered and handled with great care, and if necessary, with legal support [8]. Cost is also an obstacle. It must be overcome in order to make this technology accessible to everyone who might need it.

The bibliographic evidence shows that fertility preservation in oncological children is a subject of growing research and interest, as there are more and more patients who survive this disease and are expected to contribute to society like anyone else. There are highly complex centers in the world where preservation techniques are applied and new ways of providing it are studied, especially in groups where clinically effective ways do not yet exist.

Except for prepubertal male patients, current science is ready to offer fertility preservation to most patients whose future reproductive function may be compromised. It is important that, in light of the available evidence and with the support of the health-care managers and political authorities concerned, progress continues in the generation of knowledge and the establishment of fertility preservation units throughout the world.

## 4. Conclusions

In conclusion, the convergence of fertility preservation and pediatric oncology reflects a growing recognition of the holistic needs of long-term cancer survivors and the advancement of technology offering novel therapeutic avenues. While the historical emphasis in pediatric oncology has been on disease-free survival, a shift towards risk-adapted therapy has enabled a more nuanced approach, prioritizing both cure rates and quality of life. Parenthood emerges as a significant aspect of survivors’ well-being, necessitating the provision of fertility preservation options to all childhood cancer survivors. Effective coordination among various medical disciplines is crucial, especially given the urgency of initiating therapy. With increased awareness of the gonadotoxic effects of cancer treatments, indications for fertility preservation are carefully evaluated based on individual risk profiles. While challenges such as ethical considerations and cost barriers persist, the growing body of literature underscores the importance of continued research and the establishment of fertility preservation units worldwide. Despite current limitations in preserving fertility for prepubertal males, scientific progress offers hope for expanding access to fertility preservation for the majority of patients, underscoring the importance of ongoing collaboration and support from health-care stakeholders and policymakers to ensure equitable access to these essential services.

## Figures and Tables

**Table 1:** CED and its effects.

Female	4000–8000 mg/m^2^ CED	RR 2.74 early ovarian failure
	More than 8000 mg/m^2^ CED	RR 4 early ovarian failure
Male	4000–7500 mg/m^2^ CED	Oligozoospermia
	More than 20,000 mg/m^2^ CED	Testicular failure
RR: relative risk		

**Table 2:** Gonadal damage induced by radiation in female patients.

Infants	20.3 Gy
Girls < 10 years old	18.4 Gy
Adolescents < 20 years old	16.5 Gy

**Table 3:** Risk stratification of fertility damage by diagnosis.

High risk	>80%	Total body irradiation (TBI), pelvic or testicular radiotherapy, chemotherapy before HSCT, Hodgkin’s or non-Hodgkin’s lymphoma treated with alkylating agents and/or TBI, stage IV soft tissue sarcomas, metastatic Ewing sarcoma.
Intermediate risk	40–80%	Myeloblastic acute leukemia, neuroblastoma, stage II–III soft tissue sarcomas, osteosarcoma, non-metastatic Ewing sarcoma, hepatoblastoma, non-Hodgkin’s lymphoma, CNS tumors with radiation dose >24 Gy.
Low risk	<40%	Wilms’s tumor, lymphoblastic acute leukemia, stage I soft tissue sarcomas, retinoblastoma, CNS tumors with radiation dose <24 Gy or operated only, non-irradiated germ cell tumors.

## Data Availability

Not applicable.
